# Decision-Making under Uncertainty for the Deployment of Future Hyperconnected Networks: A Survey †

**DOI:** 10.3390/s21113791

**Published:** 2021-05-30

**Authors:** Néstor Alzate-Mejía, Germán Santos-Boada, José Roberto de Almeida-Amazonas

**Affiliations:** 1 Faculty of Engineering, Cooperative University of Colombia, Santiago de Cali 760035, Colombia; 2 Department of Computer Architecture, Universitat Politècnica de Catalunya, 08034 Barcelona, Spain; amazonas@ac.upc.edu; 3 Department of Telecommunications and Control Engineering, Escola Politécnica of the University of São Paulo, São Paulo 05508-010, Brazil

**Keywords:** uncertainty, resource management, decision-making

## Abstract

Among the several emerging dimensioning, control and deployment of future communication network paradigms stands out the human-centric characteristic that creates an intricate relationship between telematics and human activities. The hard to model dynamics of user behavior introduces new uncertainties into these systems that give rise to difficult network resource management challenges. According to this context, this work reviews several decision-making computational methods under the influence of uncertainties. This work, by means of a systematic literature review, focuses on sensor-based Internet of Things scenarios such as Smart Spaces and Industry 4.0. According to our conclusions, it is mandatory to establish a means for modeling the human behavior context in order to improve resource assignment and management.

## 1. Introduction

Data communication networks (DCN) provide the digital transmission of data between their hosts under a telecommunications infrastructure in which they share operations, administration, and maintenance. As usual, technological advances have driven the development of new approaches in DCN, giving rise to new challenges in resource management that must be tackled to take into account, for example, the data provided by end users such as in crowdsensing applications.

In traditional DCN management, performance is evaluated according to quantitative technical data, for example, packet delivery rate and throughput, among others, because DCNs were first conceived to provide a reliable and fault-tolerant infrastructure. In this approach, the data are provided by sensors, and mobile and network equipment.

However, the ever-growing interaction between individuals and their devices associated with the activity-tracking capability of such devices transforms individuals from passive to active actors in the context of DCNs [1,2,3]. Accordingly, human activities ascertain network usage profiles. Given this circumstance, the inherent uncertainty of human behavior and perception impacts DCN performance to a large extent. This new perspective is the reason for the change from infrastructure-centric networks to a Human-Centric Network (HCN) approach.

The flourishing DCN-related concept of HCN proposes the optimization of both services and network applications, focusing their decisions on the individual’s welfare. In this case, the network performance is assessed by means of quantitative and qualitative data. Individuals generate qualitative data by several means: (i) posting their opinions on web pages, (ii) using social media platforms, and (iii) logging the interactions between individuals and their devices on different platforms.

Several works have addressed these new phenomena from different points of view.

In [4], decision-making support and knowledge extraction methods that may be implemented by machine-learning techniques guided by the feedback provided by Internet of Things (IoT) and cyber-physical systems combined to cloud and fog computing have been studied.

In [5], a system implementation architecture that facilitates the interaction between entities that act based on observations of their environment called agents, in this case, human agents and machines in the manufacturing sector of the Industry 4.0 context, was described. The proposed architecture has five levels with different challenges to be mastered.

In [6], the authors proposed a logical Markovian network-based online framework for the development of voice-driven home automation systems. The objective was to enhance comfort and autonomy at home, fighting uncertainty by means of context awareness.

In [7], the authors studied the technology in networks that allows for the automation of planning, management, and optimization called Self-Organizing Networks (SON) and described a decision-making framework in which fifth-generation mobile network resources are managed by a set of machine-learning algorithms. The distinguished characteristic of their approach is the adoption of a Software-Defined Network (SDN)/Network Function Virtualization (NFV) architecture as the basis to implement decision-making technologies. The SDN are emerging communications networks that separate the control plane from the data plane to allow for interoperability, programmability, and flexibility to co-exist as features [8], and NFV allows for virtualization of network services in virtual machines, which are generally implemented individually on dedicated hardware for each service, improving scalability and agility in the deployment of new services.

It can be seen that HCN features can be advantageously used in different IoT-based applications domains such as Industry 4.0 and Smart Spaces. In theses cases, interactions between individuals and machines suggest that new computational methods combining concepts from hard and soft disciplines may improve models’ accuracies and performances as decision-making methods when uncertainty is present.

**Figure 1:**
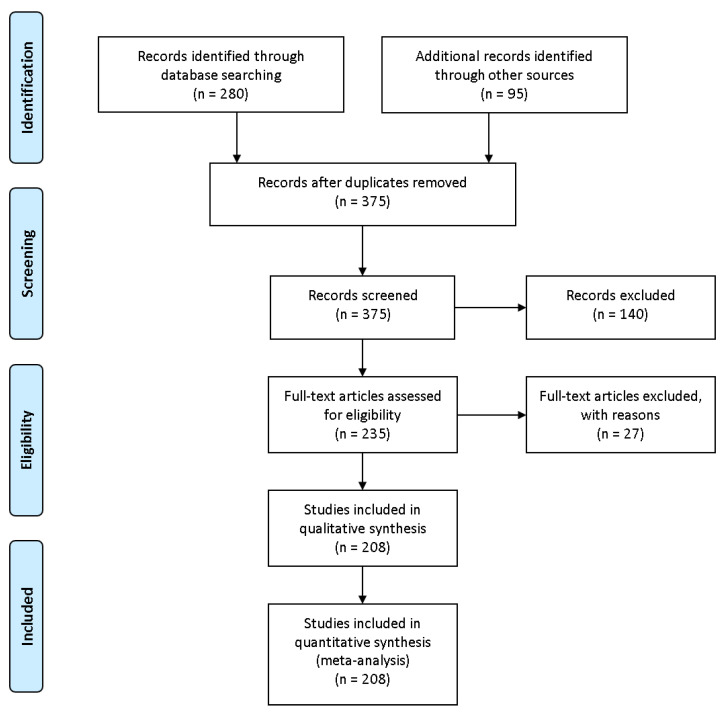
Prisma diagram.

In this work, 200+ journals, conferences articles, and technical reports were thoroughly reviewed according to the guidelines of the methodology called Preferred Reporting Items for Systematic Reviews and Meta-Analyses (PRISMA) [9] as presented in Figure 1. The peer-reviewed data were sourced from the following primary databases: IEEE, Elsevier, Springer, and MDPI.

**Figure 2:**
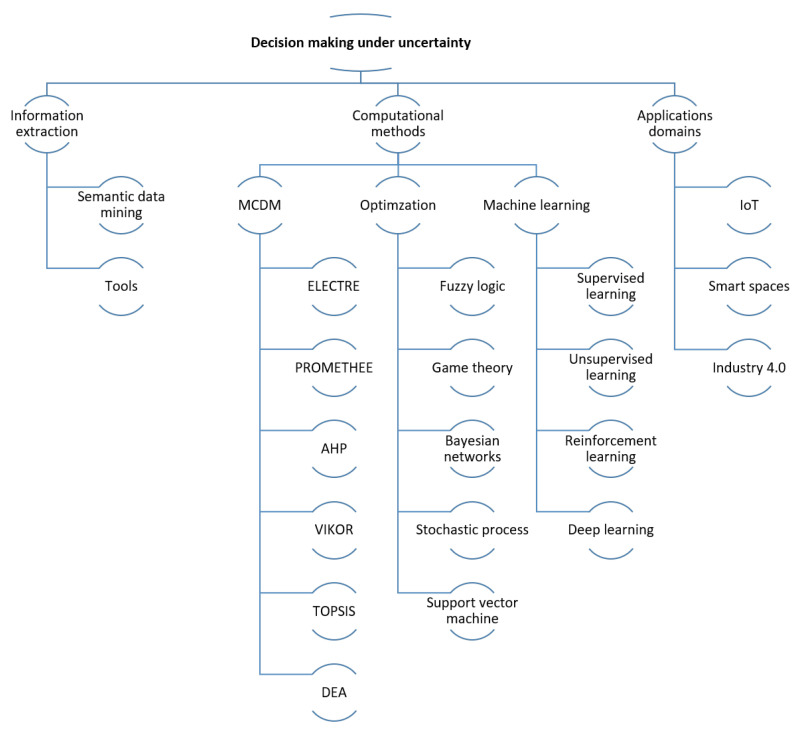
Literary review structure.

This has led us to design a search classification for our literary review, as shown in Figure 2. The inclusion and exclusion criteria were a title and abstract screening followed by a full-text and a second abstract screening process.

Three main categories were identified, namely, Information Extraction, Computational Methods, and Application Domains.

The first category, Information Extraction, has two subcategories, which are Semantic Data Mining and the Computational Tools used for extracting information.

The second category has three subcategories: Multiple Criteria for Decision-Making (MCDM), Optimization, and Machine Learning. In particular, the second subcategory, Optimization, includes developments for decision-making under uncertainty using fuzzy logic, game theory, Bayesian networks, stochastic processes, and support vector machines. On the other hand, the third subcategory contains the most significant works related to managing uncertainty for decision-making using machine learning methods, such as supervised learning, unsupervised learning, reinforcement learning, and deep learning.

The third category comprises the works of application domains that are of interest for this work, which are classified into three subcategories: IoT, Industry 4.0, and Smart Spaces.

The contributions of this work are (i) to present a systematic literature review of information extraction methods and computational methods to address decision-making processes under uncertainty, (ii) to illustrate some scenarios within the IoT application domain in which the presence of uncertainty has a great impact, and (iii) to introduce a new approach to deal with uncertainty. After this Introduction, the rest of the paper is organized according to the scheme presented in Figure 2 as follows: Section 2 reviews several information extraction methods, which represent previous and essential contributions to the decision-making process. In Section 3, a classification of various computational methods to address decision-making under uncertainty is presented. Section 4 assesses several application domains for which the implementation of these concepts is pertinent. Next, Section 6 discusses open problems and future research options. In Section 5, a novel research proposal is presented to address uncertainty. Finally, the conclusions of this work are summarized in Section 7.

## 2. Information Extraction Methods

Currently, massive Internet use, IoT, and 4G mobile telephony data transmission generate large amounts of data. Although some of these data are produced by individuals, others are produced by sensors, which register everything that happens in their environments in diverse formats, such as text, sound, images, or videos. Regarding data storage, the following options can be considered: (i) location, which may be local or cloud-hosted, and (ii) the organization of stored data, whether structured or non-structured in databases.

These features pose several technological challenges that must be addressed before extracting information from generated data, as this information provides a more comprehensive idea of what takes place. In other words, it provides context awareness, which is a determining factor for making more accurate decisions and contributes to improving decision-making processes related to resource management for HCNs.

Based on its interdisciplinary nature, data mining reviews can be performed from diverse perspectives, including databases, statistics, and machine learning [10]. However, as machine learning approaches and statistics are reviewed in further sections, this section focuses on the use of semantics as a method or procedure for extracting information as well as analyzes a different approach.

### 2.1. Semantic Data Mining

Data mining is a process used to find significant or lost information stored in large volumes of data [11]. In the information age, these processes become relevant, especially if a symbiosis is sought between human beings and machines. In this case, if both parties are able to understand the data generated by the other party, this symbiosis may prove itself beneficial. At this stage, semantics plays a significant role, wherein one must understand that “semantics is the meaning assigned to concepts and their relationships within the mind. The network of concepts and relationships is used to represent knowledge about the world, which in turn enables the cognition and perception required for the interpretation of daily experiences” [12].

Hence, in the area of semantic data mining, several studies grounded on diverse approaches have been discussed, as presented in Table 1.

IoT is a paradigm based on sensor interconnection, wherein the convergence with semantic data mining has become a popular research area [12,13,17,18,19]. For example, in [14], the authors improved resource management for wireless networks that operate cooperatively based on frameworks where semantic web techniques are applied. Furthermore, another study on the interoperability of communications in IoT systems is described in [15]. Finally, in [16], data mining challenges are assessed for cases where data are provided from different sources.

Another field related to this topic is the Semantic Web. “The Semantic Web is a Web of actionable information, i.e., information derived from data through a semantic theory for the interpreting of symbols” [28]. In this area, studies have described the diverse standpoints and execution of semantic data mining, for example, through the web [20], with association rules mining [21], based on ontology [11,22,23], and from the discovery of knowledge [24,25].

In addition, in terms of Industry 4.0, data extraction aimed at improving business profitability has been studied. For example, in [26], cyberphysical semantic systems were used as support and, in [27], intelligent logistics were applied in electronic commerce.

### 2.2. Information Economy MetaLanguage

An interesting information extraction proposal is the Information Economy Metalanguage $\left(IEML\right)$ led by Pierre Levy [29].

In fact, based on evidence of sustained increases in digital storage capacities, the omnipresence of interconnectivity based on diverse kinds of media, and the unparalleled capacity for processing, a possible question is how to take advantage of these abilities to increase our own social cognitive processes to contribute to human development. A possible reply is the collaborative and coordinated construction of a computable metalanguage without ontologies, as proposed in the book Semantic Sphere [30]: “IEML is a formal and natural language, whose semantics is computable. It is designed for use in a digital environment for data categorization, artificial intelligence and man/machine interfaces” [29].

IEML seeks to contribute to the universal identification of concepts based on a semantic coordinated system, which would allow, first, to address meanings and, second, to accurately portray how securities circulate in the general information economy.

## 3. Computational Methods for Decision-Making under Uncertainty

**Figure 3:**
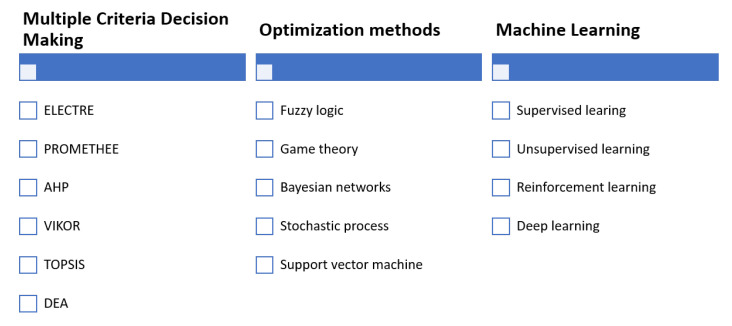
Classification of computational methods for decision-making.

Decision-making consists of selecting the most adequate alternatives from a set of possible solutions to solve a given problem in the best possible way. An agent makes decisions according to its interaction with the environment. Agents can be persons, robots, and software implemented entities [31]. This work focuses on computational decision-making systems, especially those that handle designs with both a significant degree of process automation and uncertainty. A classification of representative methods described in the literature is shown in Figure 3.

### 3.1. Multiple Criteria Decision-Making

MCDM methods assess a set of alternatives through several weighted criteria according to their relevance to the issue at hand [32]. The problem to be solved is to define how to assign appropriate weights to each of the criteria. Several MCDM methods have been extensively studied [33,34], as shown in Table 2, which may be classified depending on their academic origins, such as from European, American, and other schools.

From Europe, the following methods are worth mentioning:

- The Elimination and Choice Expressing Reality (ELECTRE) [52] eliminates non-viable solution alternatives and is usually employed along with another MCDM method in order to optimize execution times [35,36,37].
- The Preference Ranking Organization Method for Enrichment of Evaluations (PROMETHEE) [53] builds an external classification for various alternatives based on a combination of mathematical and psychological methods developing its own understanding of the problem to help agents choose the option that best serves their purpose. This method has been evaluated by different domains such as infrastructure construction [38], the electric power sector [39,40], and engineering decision-making [41], among others.

The North American school proposes the following:

- The Analytic Hierarchy Process (AHP) [54] combines conflicting physical and psychological elements based on appraisals and assessments to manage complex decisions. In [42,43], decision-making is handled under uncertainty, while in [44], it is based on subjective product recommendations from consumers.

From other origins, we may cite the following:

- The Multicriteria Optimization and Compromise Solution (VIKOR) [55] aims at determining the best possible solution when dealing with conflicting options or with different methods of measurement. In [45], VIKOR is combined with other techniques to assess feelings in social media; in [46], group decision-making processes are implemented; and in [47], it is used in the evaluation of airline service quality.
- The Technique for Order of Preference by Similarity to Ideal Solution (TOPSIS) [56] aims at finding an alternative solution using the shortest and longest Euclidean distance from the optimal positive solution and the optimal negative solution, respectively. This method is usually enhanced by additional algorithms, as shown in [48,49,50].
- The Data Envelopment Analysis (DEA) [57] assesses the relative efficiencies of comparable entity sets by solving a series of mathematical programming models [51].

### 3.2. Optimization Methods

Optimization methods aim at maximizing profits or minimizing risks in the decision-making process by ascertaining the option that best solves the problem pursuant based on the target function. Consequently, research focused on optimizing and managing decision-making uncertainty is classified as per our literature review findings, as shown in Table 3.

#### 3.2.1. Fuzzy Logic

Fuzzy logic creates a mathematical framework that may be adapted to actual complex problems that add uncertainty to human cognitive processes. Several purposes can be achieved by this capability when using different fuzzy logic computational methods as described below:

Fuzzy Set: In contrast to set theory, where each object has a binary value (member or not member), the values of fuzzy set objects can range from 0 to 1 to reflect inaccurate or uncertain conditions. For this method, several extensions exist, as listed below:

Interval-Valued Fuzzy Sets: This method intuitively addresses uncertainty and inaccuracies whenever there is no accurate knowledge of the function to be assessed, as in [58,59,61,62,71].

Type-1 Fuzzy Set: “A type-1 fuzzy set A is a set function on universe X into $\left[0,1\right]$, possibly constrained to belong to a family such as continuous functions, i.e., $\mu_{A}:X\to \left[0,1\right]$” [69].

Type-2 Fuzzy Set: Unlike the Type 1 method, which is a special case of a Type-2 method, this method is regarded as a way to increase the fuzziness of a relation. Based on its characteristics, it is an ideal method for addressing linguistic uncertainty, which may arise since words have different meanings for different people, as described in [45,60,70].

Vague Set: This method estimates the lower and upper probability bounds that determine whether any given element is a member of the set, and it is useful for finding inconsistencies in interval assignments to the Boolean expressions used in sets. Its advantages are listed in [63].

Rough Set: This method is applied when dealing with problems with incomplete information. For these purposes, this method removes irrelevant data from the approximate set without affecting the original system and generates decision rules to complete the remaining values. In addition, References [38,64,65,66] discuss the wide range of fields to which this method may be applied.

Mamdani-type: It is a fuzzy inference method that calculates an output value for an input value, and it is used in cases of information uncertainty, as described in [67,85].

#### 3.2.2. Game Theory

This is a mathematical tool applied to human decision-making. This theory assumes that individuals are rational and have conflicting positions to address how they attempt to maximize their benefits when interacting under defined rules. Consequently, new methods have been proposed to address uncertainty, as described in [72,73,74,75].

#### 3.2.3. Bayesian Networks

Bayesian networks use directed cyclic graphs to distribute probabilities among the corresponding random variables for each node. Their advantage relies on their ability to reduce the number of parameters required to determine a joint probability distribution. However, as nodes are not connected, finding a path from one variable to another may be not feasible. In fact, there are several algorithms that create inferences to learn about Bayesian networks, which may be used to overcome uncertainty in decision-making processes, as in [76,77,78,79,80].

#### 3.2.4. Stochastic Process

Stochastic processes include a set of random variables associated with one another wherein one of these variables usually represents time. Each random variable has its own probability function and variables may be correlated to each other. These random features have been used to cope with uncertainty in different fields, as in [6,81,82].

#### 3.2.5. Support Vector Machine

These are several supervised learning algorithms applied to classification or regression problems. These algorithms usually produce accurate results, at high computational costs, which may even improve if data are scattered. Therefore, this method has been used to treat uncertainty in studies, such as [83,84].

### 3.3. Machine Learning

**Figure 4:**
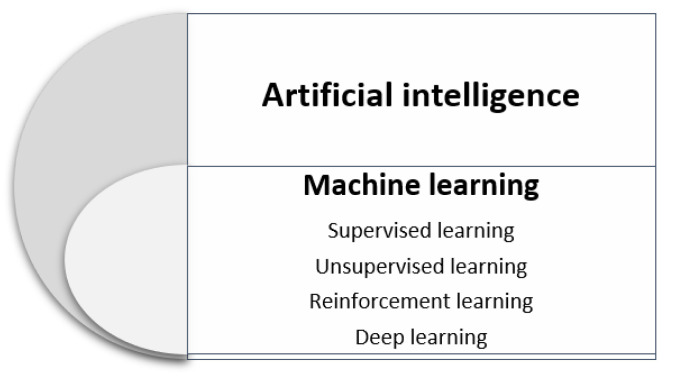
Classification of machine-learning methods.

Machine learning, a subfield of artificial intelligence, is focused on developing algorithms that provide computers with learning capabilities about their environment, with the purpose of improving and adapting themselves to the challenges faced. At present, due to the large amount of data generated by several fields, such as telecommunications, energy, transportation, finance, and health, among others, this capacity is commonly used whenever data-based solutions are needed [86]. Machine learning algorithms have been classified as supervised, unsupervised, reinforcement, and deep learning methods, as described in Figure 4.

#### 3.3.1. Supervised Learning

In this method, learning takes place through a training process using labeled datasets. This method is commonly used when predicting something that is already known. Table 4 lists the works wherein this decision-making method under uncertainty has been applied.

- Telecommunications. In this field, supervised learning has been used based on factors such as QoS and automatic configuration. For example, QoS has been used to create resource appraisal and classification models for IoT [87] or for any type of network [88]. It has also been used as a retroactive measure to tailor service selection [89] and for decreasing the time required by virtual machine migration processes through WAN links [90]. In addition, in terms of automatic configuration processes, some studies address 5G networks [7,92] and elastic cloud systems [91].
- Energy. Several types of problems of this field have been solved by applying the supervised learning method, such as preventive planning of uncertain operations in power systems [93]; by selecting the best maintenance route to minimize operational costs when power grid failures occur [94]; by predicting nuclear power system behavior [95]; and by selecting the best location for wind turbines based on economic, regulatory, and social factors [96].
- Transport. In this field, several studies focus on process improvement, such as speed and accuracy in lane changing maneuvers when driving on highways [97], driving terrestrial vehicles on rural roads [98], robots learning routes through linguistic decision trees [99], and methods used in biped robot walking processes [100,101].
- Enhanced decisions. These studies focus on improving the performance of decision-making results by choosing selection mechanisms in complex negotiation scenarios [102], by considering environmental awareness when dealing with critical complex systems processes under supervision [103], by assessing the advantages of combining the learning process with multiple agents and weighted strategies [104], and by suggesting two complementary stages that would exist between machine learning and support vector machines [105].
- Complex systems. These problems are computationally difficult to model, especially if related to human behavior in cooperative work, as discussed in [106,107,108,109].
- Optimization problems. When it is not feasible to find an optimal solution for uncertain problems, a solution that satisfies problem constraints may be selected, as described in [84,110,111,112,113,114].

#### 3.3.2. Unsupervised Learning

In this method, non-labeled input data patterns are learned without any corresponding output variables. As unsupervised learning is applied when the target in unknown, its algorithms attempt to model the structure that underlies the data. Table 5 lists the main works related to this method.

- Transport. When applying this method to this field, research is centered on obstacle management for autonomous driving from various approaches. For example, in terms of the epistemic uncertainty of images [115], semantic segmentation methods achieve high inference classification accuracy in object recognition within interior spaces [116] or in different other additional challenges, as listed in [117].
- Health. The works proposed in this field are aimed at improving unsupervised learning accuracy and solution times, as denoted in [118], which discusses liver fibrosis diagnoses. Furthermore, in [119], this method was used to customize patient therapy processes and, in [120], it was used to diagnose complex vision pathologies.
- Business decisions. In this field, research works focus on earning profits by selecting the best decisions, as described in [121], where feeling assessments are combined with share price volatility. In addition, an evaluation of industrial systems in terms of sustainability through hard-to-find indicators was presented in [83]. Another study discussed learning from previous decisions through comparative evaluation processes [122]. This method was also applied in the education sector to assign students to a company depending on their skills [123] and to improve manager actions at universities [124].
- Dealing with uncertainty. Studies seeking to solve unsupervised learning problems dealing with uncertainty use different approaches, such as in battleground decision-making [125]. In geology, it is used for water- and oil-flow systems [126]; in gambling, its is used for the Khun poker game [127]; and it can be used when merely facing the uncertainty of applying this method to any type of problem, such as in [128,129].

#### 3.3.3. Reinforcement Learning

Reinforced learning is used to train systems based on a sequence of punishments and rewards. Therefore, sample datasets are not used since the system learns based on trial and error. This method was first applied in video games, where the agent can go through all possibilities until the optimal solution is found. At present, it is widely applied to complex fields, such as autonomous driving. Several articles explore using this method in various fields, as listed in Table 6.

- Telecommunications. Based on this method, several topics have been addressed within this field, such as resource management, particularly regarding power consumption for large-scale IoT applications [130]. In [131], a framework was proposed based on reinforcement learning for the SDN control plane to intelligently manage uncertainty in 5G networks. In routing, as in [132], the authors planned to prevent gateway bottlenecks by identifying the best path for reaching the best gateway through reinforcement learning techniques.
- Energy. For energy companies, decision-making is complicated due to the high level of uncertainty that exists. For these purposes, there are proposals, as in [133], seeking to balance supply and demand in real time in Smart energy markets, or as in [134], which improves previous energy market negotiations by adapting techniques such as Q-learning.
- Transport. Based on reinforcement learning, specifically in this field, there are contributions on issues such as autonomous driving [135,136,137,140] and experience management [138,139].
- Optimization problems. By combining techniques with the reinforcement learning method, applications have been optimized, such as computational costs [141], data noise management [142], scalable solutions in terms of time and scale [143], or the adjustment of evaluation functions [144,145].

#### 3.3.4. Deep Learning

This machine learning method mimics human learning processes. By using an interactive simulation process, this is accomplished with labeled and unstructured data, and ultimately, a statistical output model is developed. In fact, this process can be conducted without requiring supervision from the developer as it facilitates operational work and prevents errors that may have been induced. However, the accuracy of the model relies on the existence of a large amount of data to train the machine, which, in turn, requires high processing capacity for running simulations. Nevertheless, technological advances and their increasing interaction with human beings have fostered particular interest within the scientific community regarding deep learning in certain fields, as described in Table 7.

- Telecommunications. In this field, research has been conducted on topics such as resource allocation management in portable sensors [146], wireless networks [147], low-power devices [148], and cognitive radio networks [149]. Another important topic is SDN application in Smart cities [150].
- Human Behavior. On this subject, different research approaches have been used. For example, as a first approach, human behavior prediction models have been developed, either when decision-making is affected by peer pressure or by the inference of human activities based on short videos [151]. The approach ranges from psychological perspectives to assessing decision-making abstraction in human beings, both in regular contexts [152] or with imperfect information [153].
- Uncertainty. Through deep learning, uncertainty challenges have been studied under different approaches. Some examples are uncertainty problems due to subjective opinions in heterogeneous networks [154]; in military scenarios, the uncertainty in unmanned aerial combat vehicle decisions [155]; uncertainty modeling in real time for the relocation of automatic visual systems [156,162]; issues when dealing with missing data due to the calculation of uncertainty based on the remaining training dataset [157]; and a random estimation method to calculate uncertainty in object detection for applications that require reliable decisions [163]. Another area is decision-making under uncertainty, such as whenever assessing complex structures [158] or emotions are expressed in texts [159]. Finally, the work associated with taking risks under uncertainty has been mentioned, either as analyzed from the perspective of video games, as in [160], or in financial forecasts for customers [161].

## 4. Internet of Things Application Domain

Internet of Things emerged in the 1990s as a new concept from the possibilities offered by the new communications technologies, for example, the Radio-Frequency Identification (RFID). Kevin Ashton introduced the term IoT at a presentation in 1999 [164]. From then on, the International Telecommunications Union (ITU), the Institute of Electrical and Electronic Engineers (IEEE), the European Telecommunications Standards Institute (ETSI), and the Internet Engineering Task Force (IETF), among others, have been working on its standardization, having yet to reach a universally accepted IoT definition. In our opinion, the definition provided by the EU-funded project Coordination and Support Action for Global RFID-Related Activities and Standardization (CASAGRAS) captures the essential characterisitics of IoT: “A global network infrastructure, linking physical and virtual objects through the exploitation of data capture and communication capabilities. This infrastructure includes existing and evolving Internet and network developments. It will offer specific object-identification, sensor and connection capability as the basis for the development of independent cooperative services and applications. These will be characterized by a high degree of autonomous data capture, event transfer, network connectivity, and interoperability” [165].

### 4.1. Mobile Wireless Sensor Network

**Figure 5:**
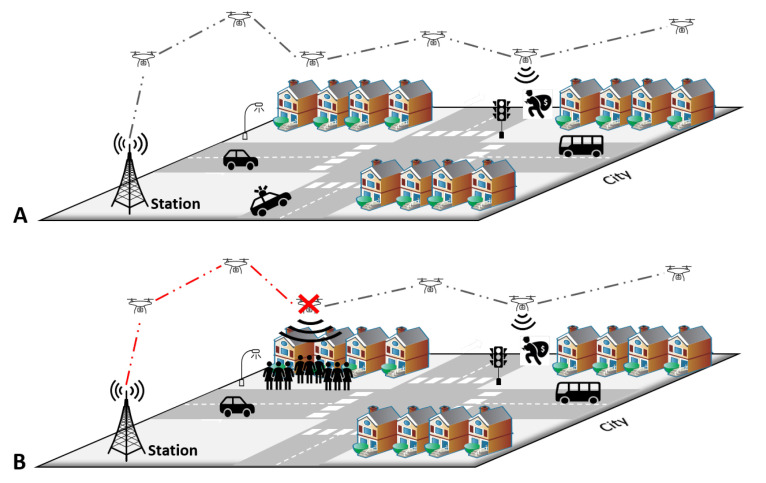
A MWSN scenario. (**A**) Illustration depicting a scenario without uncertainty, (**B**) system failure due to introduction of human uncertainty.

A Mobile Wireless Sensor Network (MWSN) shows how uncertainty is present within an IoT network. Let us consider the case depicted in Figure 5. MWSN resources are limited in terms of processing power, storage capacity, and energy availability. In this scenario, mobile wireless sensors installed on drones monitor and identify intruders in specific neighborhood areas. They can communicate directly with each other without using the infrastructure of a deployed physical network. Figure 5A shows a late-night example, at hours when it is unlikely that people are out in the streets. A sensor detects an intruder, and based on the context of that moment, the only possible route to the sink node is found and an alarm message to the control station is sent.

Nonetheless, the movement of the drones may lead the routes becoming unfeasible because one or more sensors are outside the coverage range. Additionally, hardware failures and lack of processing power may also disrupt the route. Figure 5B exhibits a case in which one of the drones goes offline because the required resources exceed its capacity. This may happen due to a catastrophic occurrence that may compel people to leave a region hurriedly. This example shows the importance of context-based decision-making algorithms in the presence of always possible, although unlikely, uncertain events.

### 4.2. Smart Spaces

The consulting and IT research company Gartner declared Smart Spaces as one of the top ten technological trends back in 2019. According to Gartner a Smart Space is “a physical or digital environment in which humans and technology-enabled systems interact in increasingly open, connected, coordinated and intelligent ecosystems” [166].

**Figure 6:**
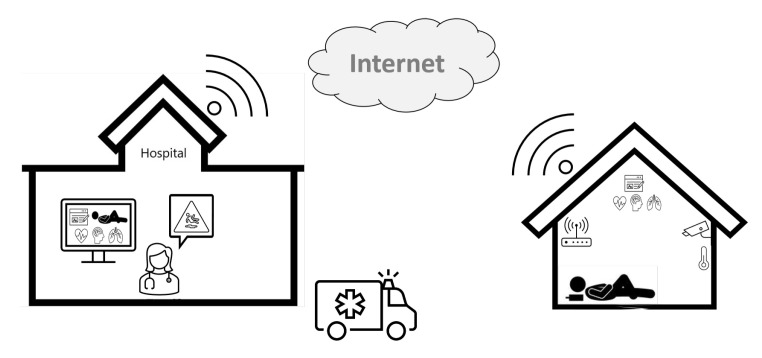
A Smart Space scenario.

Figure 6 illustrates an assisted living scenario of a healthcare application to monitor daily activities and vital signs of the patient. As health may also be influenced by changes in light, temperature, humidity, and noise, these variables should also be monitored and controlled. In this case, the house is equipped with the necessary sensors and devices to control the environment and to alert a nearby hospital if needed. The application’s objective is to provide immediate assistance either by making a phone call or by sending paramedics. The patient’s social media is also monitored to provide additional data and context-related information to be used alongside those extracted from the sensors. However, some situations may occur for which it is not clear what is going on. For example, let us assume that, because of back pain, the patient decides to lay on the floor and falls sleep. Depending on when the images or videos provided by the cameras are seen or processed, it may appear that the patient has fainted or suffered an accident. If the patient is having a nightmare, their heart rate may be altered. If the images/video cannot be interpreted correctly, the devices may send a false alarm message. This example illustrates once more the importance of uncertainty management by automatic decision-making methods.

### 4.3. Industry 4.0

Beginning with the first industrial revolution, all disruptive technological advances have marked an industrial evolution. The lives of people, society, and organizations have been transformed by these developments. Nowadays, we are advancing toward the fourth industrial revolution, which originated from the term Industry 4.0 used by the federal German government during the presentation of its Industrie 4.0 initiative in 2011. The Smart symbiosis between machines and human beings is the technological disruption that was intended by Industry 4.0, which is spurred by information and communication technologies [167].

To examine the impact of uncertainty on Industry 4.0, let us consider the case of a call center that provides online customer services for a company in the context of an Industry 4.0 scenario using the resources of a mobile phone company. The objective of these centers is to manage as many customers as possible. As the network resources both in terms of available telephone lines and bandwidth are limited, it is of paramount importance to monitor and control the holding time of user calls. The call center has a Customer Relationship Management (CRM) system to monitor, record, track, and forecast events. Furthermore, the CRM builds up a profile of each user. After receiving an explicit authorization, the system is able to record data extracted from social media databases, cell phones, or any other means. These data can be used as ancillary information to process the user requests received by telephone calls, chats, and emails in a more effective way. Notwithstanding, if the information derived from the raw data is not accurate and contextualized, the actual time spent providing an answer to a call may increase waste of resources in the network infrastructure.

For example, let us consider the scenario where some users have been chosen to respond to a survey with a maximum timeframe to identify their preferences to customize a given product within the context of an Industry 4.0 environment. Users were selected among those that are declared (i) to be highly satisfied with the products of the company sponsoring the survey; (ii) to be young, as it is assumed that they can better handle new technologies; (iii) to have a high income; (iv) to live in urban areas; and (v) to have achieved a minimum academic degree. Surprisingly, some of the users that fulfill the predefined criteria fail to complete the survey within the time limits due to conditions not captured by the extracted data. For example, some of them may undergo some cognitive, hearing, vision, or speech impairment and others may try to answer the survey while performing some other task at the same time.

Industry 4.0 features may lead to deep changes and transformations in terms of labor rights, gender and social inequality, and new business models. However, this new scenario has to be supported by high-quality human-centered networks and computational intelligence able to handle the uncertainty introduced by people behavior. As far as communication networks challenges are concerned, according to the reviewed works, the proposals focus on SDN. Industry 4.0 uses SDNs to implement network virtualization [168], Ethernet network metrics [169], cloud manufacturing [170], cybersecurity [171], or resource allocation and information exchange for IoT or Industry 4.0 [172,173].

## 5. Radical New Approach to Deal with Uncertainty in HCNs

User-related and generated data (for simplicity referred just as user data) constitute the basis of almost all social applications based on interactive media technologies. However, in many situations of interest, mainly those that can be described as emotionally stressful, user data lack reliability. The reasons for the lack of reliability are manifold, going from simple mistakes when describing a situation to a distorted understanding of reality due to an emotionally impaired perception.

A scenario that accurately exemplifies the long-term vision of this new approach application can be illustrated in disaster management. In a hypothetical event of a car accident on a frequented highway, where many other cars pass by, we can imagine many social media posts describing details about it before any truly reliable information (e.g., the police arriving) is conveyed. In such a case, if the high volume of uncertain quality user-based information is instantly filtered so that a reliable source that, e.g., accurately reports the degree of passenger injury severity, is identified, the timely arrival of an ambulance could have a life-saving impact. Of course, the above is a nontrivial task as it implies mechanisms which are yet non-existent; it is certain though that an interdisciplinary approach is necessary to capture the diverse aspects of methods of human social expressions of perception in terms of data and to model it in a formal way in order to conceptualize such mechanisms and to infer the reliability of the information and knowledge that can be acquired from such data.

Due to this lack of reliability associated to user data, interactive media technologies-based applications that depend on the characterization and prediction of the information and knowledge acquired from such data may be severely impaired and are rarely adopted by the actors involved in real-life scenarios.

We conjecture that psychoanalysis theories may be used to objectively measure the reliability of the user’s discourse. From now on, this new approach is referred to as Psychoanalysis-Driven Computing (PDC).

User data characterization and processing in the form of text and speech are the input of PDC. Apart from traditional user data, such as the aforementioned, PDC considers new and emerging media type issues, such as crowdsourced data that are collected using public participation. In particular, by identifying specific patterns in user data regarding an individual, a group of people, or even a crowd, one can associate the user’s data stream in the context of the structural entities of the Lacanian theory, namely, the Lacanian Discourses. The Four Discourses theory constitutes an attempt of formalization of the different ways people relate to each other and the economy of knowledge and enjoyment in social relationships. The Lacanian framework defines a more complex representation of the roles assumed by two interacting parties, formulating four discrete discourse types [174,175,176]: Discourse of the Master—struggle for mastery/domination/penetration; Discourse of the University—provision and worship of “objective” knowledge, usually in the unacknowledged service of some external master discourse; Discourse of the Hysteric—symptoms embodying and revealing resistance to the prevailing master discourse; and Discourse of the Analyst—deliberate subversion of the prevailing master discourse.

Later, Lacan defined an additional fifth discourse, which is also considered: the discourse of the Capitalist [177], where the subject is commanded to enjoy commodities.

**Figure 7:**
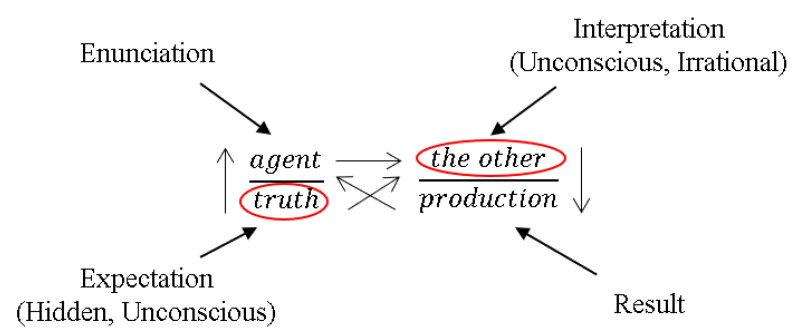
The four places of the discourse: the “agent”, the giver of the discourse; the “other”, the one to whom the discourse is addressed; under the message of the agent is hidden the “truth”, which is masked by the official statement; and hidden under the other is the “production”, or what the agent gets out of the relationship.

Each discourse is represented by an algorithm, containing four elements distributed in the four places of Lacan’s formal representation of an interaction (Figure 7). It is possible to draw a parallel between the terms of a discourse and the components of a communication process, in such a way that the dynamics of a given discourse, i.e., the internal relations between elements arranged in different places, can serve to characterize the dynamics of a given media process.

Precisely, the idea is that, by distinguishing the discourse type, the “truth” status of an enunciation can be qualified, i.e., it is the formal characteristics of the discourse that inform whether the truth is based on authority, on documented sources, on the needs of the speaker, or on provocation. For instance, in a hysteric discourse, the truth is strictly singular and reflects an individual experience; in a master discourse, the truth has been decreed by authority; in a university discourse, the truth has been documented by elements that act as sources of authority (e.g., objective counting); and finally, in an analyst discourse, the statement is to be heard as a question (or a provocation) in the hope of obtaining a reaction on that point. Nonetheless, mapping of an interaction with the Lacanian Discourses does not happen in an absolute way; instead, percentages of association with each of the discourses are derived, with the sum of all being 100%.

In this work, we propose the reconcilation of two fields that are both exceptional to the scientific discourse today: modern computing theory and psychoanalysis.

Tools coming from fields such as data mining, semantic and linguistics, or machine learning are necessary for analyzing the different kinds of user-generated language. A trained psychoanalyst can detect the above signifiers based on semantical analysis of the language; by using digital analysis of user data, we aim to abstract the methodology of a psychoanalyst in order to identify particularities in the speech signal in a purely computational manner. Given enough annotated data, we envision that, using machine learning, pattern recognition techniques, and games theory, such methodologies can be generalized to other typess of support, such as interactive discussion in social networks.

More specifically, to address implicit, hidden (i.e., unconscious) user expectations, emotions, attitudes, and interpretations of exchanged information, we use a variety of Computer Science (CS) methods such as the following:

- Text mining and semantic analysis techniques, towards semantically rich representations of exchanged user texts;
- Graph-based representations of user data and relevant network metrics (such as centrality), towards identifying key user behavior types and patterns;
- Markovian models (Hidden Markov chains, dynamic graphs processes, information spreading, etc.) [178] to capture inherent dynamics and complex impacts of user data and Partially Observable Markov Decision Process (POMDP) to cope with the inherent uncertainty due to unreliable and/or incomplete information [179];
- Bayesian Games [180,181,182,183], since in many situations, decision makers are not perfectly informed about the characteristics of others;
- Non-Markovian models (to capture special dependency on the current state) such as Martingales [184];
- Key algorithmic methodologies (primarily machine learning and cognitive reasoning) to characterize the user data, their reliability level, and the associated discourse types; and
- Game theory methods, which can contribute to behavior prediction of rational individuals (and the society as a whole) [185,186,187,188]. It is worth noting that variants of game theory models (including penalties) can even address (and perhaps mitigate) “irrational” behavior, due to, e.g., to hidden, latent, or even unconscious mechanisms in one’s behavior and actions.

It can be argued how the Lacanian Discourse approach can be extended to groups with more than two interacting parties. The answer to this concern comes from recognizing that psychoanalysis is a general framework for the interpretation of situations expressed in any format and by any number of people. Since its development by Freud, it has been stated and shown that it can be used to interpret works of art [189], to analyze social situations [190,191], or to conjecture about the future of civilization [192,193]. These are just a few examples of Freud’s works applying psychoanalysis techniques out of the psychoanalytical setting of a patient and an analyst.

The Lacanian Discourses are a formal framework to apply psychoanalytical concepts to interpret any real-life situation. The aforementioned Freud’s works can be used as a reference model to interpret works of art, social situations, etc.

From the computational perspective, game-theoretic methods are applied to promote rational behavior of people interacting with each other.

Nevertheless, the association of an interaction with one of the discourses is not an easy task and has to be dealt with cautiously. An example that illustrates how a single media phenomenon can be seen from various angles according to discourse theory is provided by [194]: “When Google scans the Internet collecting information from each site, we are in the discourse of university. When it meets our demand providing results, we are in the discourse of hysteria. When we deify it, we are in the discourse of the master. When it computes our data and customizes the results it offers us, as if it knew us, knew our preferences and anticipate what we want, we are in the discourse of capitalism”. Therefore, it is paramount that the context is well defined before proceeding to association, so that the stakeholders of the discourse are clearly identified.

Context depends on the type of application. Kknowledge about the application implies knowledge of the context and establishes the contextual elements to be considered. The method proposed by PDC is general enough to be applied to any context.

To complement the above methodologies, user data are also analyzed independently in terms of coherence, using text mining techniques, which again partly aim to abstract the methodology of a psychoanalyst in a session but, this time, with respect to the theory of primary process mentation.

It will be important to combine the Lacanian Discourse type with the primary process index: it is more specifically in the Master and in the Hysterical Discourses that high primary process scores invalidate the reliability of the information conveyed in the discourse. Repetition in either the University or in the Analyst Discourses does not necessarily need to invalidate the reliability of the information (in the University Discourse, it might simply denote the wish to repeat the same, valid information, while in the analyst discourse, the repeated attempt is used to provoke a reaction). Thus, based on the above, the computational goal and challenge is to define methods to estimate these primary process indexes and to produce an associativity coefficient based solely on user data. By doing so, we acquire a metric that reflects the variance in a user’s language and that can serve as a global score of “stability” in producing data that can be used to acquire reliable information and knowledge.

Therefore, besides the Lacanian Discourses, the α and β concepts introduced by Wilfried R. Bion (1897–1979) [195,196] are used. In short, α-elements may be considered as the quanta of creative thoughts while β-elements are the quanta of useless thoughts. These elements can prove helpful when formalizing the underlying rules of a subject’s relationships with its social context, with the aim of producing quantifiable representations. For instance, the efficacy of a Web Learning Environment was analyzed in [197,198] using Bion’s concepts adapted to a remote learning session.

In summary, PDC proposes the building of a theoretical framework that allows for the characterization of user input in social interactions in terms of reliability in a tangible way by providing corresponding reliability coefficients. This occurs in a systematic and automatized way, starting from various forms of user data as input and by combining quantitative association to the Lacanian Discourses with extracted associativity coefficients, in order to produce a single reliability coefficient. This is undoubtedly a nontrivial task, as currently, there is no theoretical framework for quantitative association to the Lacanian Discourses or for such reliability coefficients. Therefore, during the PDC workflow, we study and develop mathematical models to define the theoretical background to serve as a basis for the above framework, providing methods and methodologies to automatically calculate the aforementioned associations and to produce the desired coefficients. In addition, a corresponding evaluation framework is established to provide quantifiable metrics for estimating the accuracy of the developed tools.

To illustrate how the PDC approach can be applied to evaluate user data reliability, consider the following hypothetical and extremely simple scenario illustrated in Table 8: a car accident scene and two different persons (“users”) witnessing it. the reactions and feelings of the two users are summarized below:

In this simple case, it is easily seen that the data provided by User 2 is most likely much more reliable than those provided by User 1. To arrive objectively at this conclusion, we introduce $I_{m}$, $I_{u}$, $I_{h}$, and $I_{a}$ as indices corresponding to each one of the four basic Lacanian Discourses; their aim is measuring closeness to each discourse type as a result of some piece of data provided by a user. Additionally, let α and β be indices corresponding to the α and β elements in Bion’s theory. An indicative assignment of values to these indices, based on data provided by the users is given in Table 9 (their mean values are also calculated at the end).

**Figure 8:**
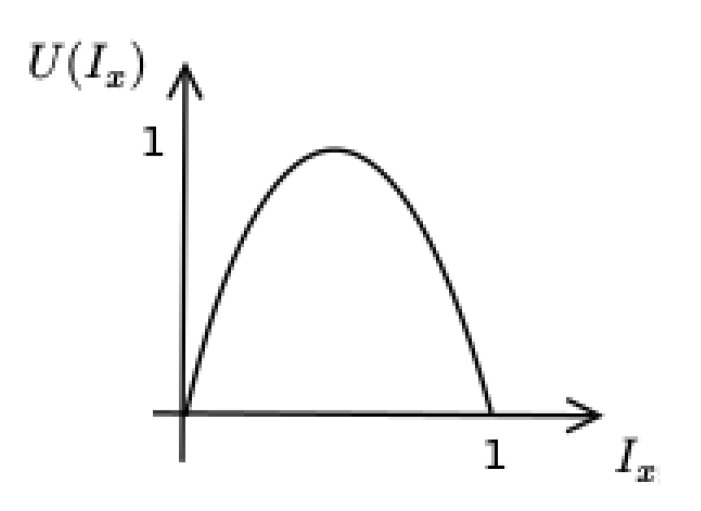
Adopted utility function for any Lacanian index.

The utility function for any Lacanian index $I_{x}$ given by $U\left(I_{x}\right)=-4{\left(I_{x}\right)}^{2}+4I_{x}$ is chosen to promote a “balanced” discourse in the sense that both the complete lack of a discourse type and an exaggerated discourse dominance orrespond to an extreme, incoherent discourse. The form of $U(I_{x})$ can be seen in Figure 8.

Furthermore, we assume that utilities of individual indices are additive; thus, a multi-utility function (MUF) formulating closeness to the Lacanian Discourses may be given by the following:

$$U\left(I_{m},I_{u},I_{h},I_{a}\right)=\frac{1}{4}\left[U\left(I_{m}\right)+U\left(I_{u}\right)+U\left(I_{h}\right)+U\left(I_{a}\right)\right]$$

In a similar manner, we may formulate the associativity coefficient as follows:

$$C_{A}=\frac{1}{n}\sum_{i=1}^{n}\left(\alpha_{i}-\beta_{i}\right)$$

where *n* is the number of pieces of user data.

Applying these formulations to the specific example, we get the following:

- User 1: $C_{R}=0.255-0.500=-0.245$
- User 2: $C_{R}=0.46875+1=1.46875$

As we can see, the information acquired from User 2 is much more reliable than the information acquired by User 1.

As mentioned before, this is just a preliminary, indicative derivation aiming to exemplify our approach. Our approach is significantly enriched during the development of the PDC project, when we further study and evaluate different utility functions (potentially context-dependent) and diverse formulas for the two fundamental coefficients (reliability and associativity).

## 6. Discussion

**Figure 9:**
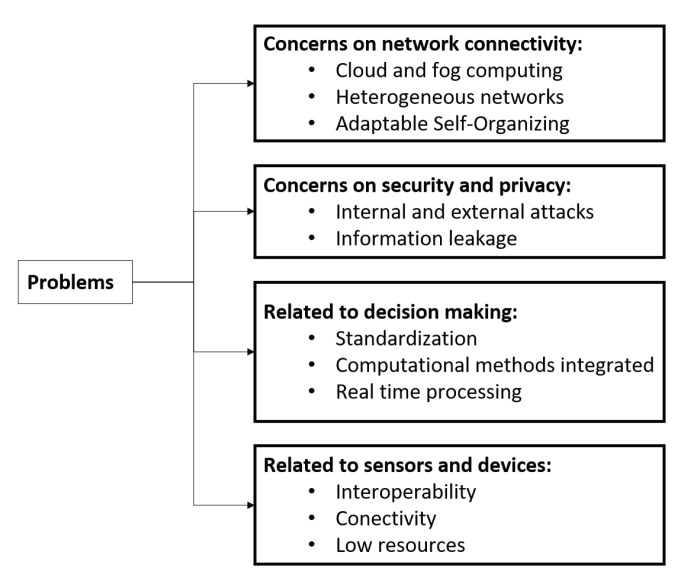
Open problem categories.

The increased interweaving of networks and systems with human activities creates new development possibilities within several contexts. However, this brings about several challenges in the triad assessed in this work, such as the integration of decision-making under uncertainty, network resource management, and HCN. At this stage, we describe the main open research problems arising within this context, classified as presented in Figure 9.

- Concerns in network connectivity. Mobility is one of the main characteristics of modern networks. However, depending on the technology, mobility may face challenges, such as radio spectrum reservations and allocation, bandwidth allocation, transfers, and routings. If communications are between heterogeneous networks, these situations may become quite complicated. Therefore, standardized self-organization mechanisms are required for the infrastructure to adjust to the constant changes caused by mobility and user demands, regardless of technology. However, cloud computing support provides greater availability and possibility for distributed and online processes to be implemented. Remote processes may be managed and monitored in manufacturing, medical care, or surveillance, among others, through their interaction with IoT. Nevertheless, in applications wherein response times are critical, this technology must be correctly assessed to prevent proper operations from being disrupted. Moreover, in contrast to cloud computing, fog computing uses a decentralized infrastructure that adapts to the specific nature of HCNs since devices can be distributed in several regions. Consequently, with fog computing, the data produced by devices is processed closer to the places where they are generated, which means that they are not uploaded over long distances to any cloud, thus improving the performance of the services offered by HCNs while decreasing response and reaction times. However, as fog computing continues to face several new challenges, such as business models, security, privacy, and scalability, further research on these areas may be required [4].
- Concerns about security and privacy. Security risks can have economic, environmental, and organizational consequences that may be related to personal, social, or industrial environments. Even people’s lives may face some type of risk. Malware that impacts devices connected to networks may reduce DCN performance and may compromise package delivery to such an extent that the whole network may collapse, among other examples. Attacks from malware, such as Mirai, take control over IoT network devices, such as IP cameras, printers, routers, sensors, and others, to carry out a distributed denial of service attack. The Mirai attack was considered the most devastating in history because it affected around 164 countries and blocked Dyn, one of the most important domain name system service providers for worldwide companies. The attack affected application services provided by companies such as WhatsApp, Github, Twitter, PayPal, and Spotify. Another consequence may be the interception or modification of personal or business information. Issues related to cybersecurity, where the authorization and authentication of sensors, devices, and actuators, are critical for securing trust in HCN operations.
- Elements related to decision-making. It is somewhat challenging to make decisions on cross-cutting issues such as HCN management due to the different types of resources used, such as heterogeneous networks, several types of sensors and devices, and the vast number of data collection sources, among others. Based on these reasons, reaching agreements on standards is a continuous improvement issue [199,200]. Furthermore, integrating decision-making and machine learning is an exciting matter due to the large amount of data, processing capacities, and the range of techniques that must adjust to needs under data uncertainty. In fact, this may even include exploring diverse possible potential integrations between MCDM methods and machine learning for each different phase of the decision-making process [94,201,202]. Another challenge faced is real-time decision-making processing because decisions are effective only if made in real time [203]. This involves several factors that require further research, such as data accuracy, low response times, distributed processing, and security methods [204].
- Challenges related to sensors and devices. Regarding electronic devices, they take samples and report the behavior of environment variables or of the individuals for whom these variables were created. In both instances, problems arise in terms of access conditions to power grids or communications networks [205,206], and especially for wireless sensors, which, in general, exhibit decreased processing resources, battery autonomy, wireless range or security [207]. These constraints contrast with the demands for resources needed when applying machine learning techniques, which implies that identifying computationally efficient strategies is an essential component. Various device manufacturers are another issue, since each has its own input and output data formats, protocols, and interfaces and this hinders interoperability and smooth operations.

We identify, at least, the following future research directions:

- IoT massification and the deployment of the 5G network will cause high network densification, which brings about the need to examine new routing protocols that may support constant changes in user contexts. This means that these protocols may use context information whenever moment priorities change, employing the required resources to meet new objectives.
- The large level of ubiquity and information exchange among users and systems will facilitate security threats sustained by artificial intelligence. Therefore, there is an urgent need to set up policies and measures to protect personal information.
- HCNs can potentially generate large amounts of data due to the integration and interconnection of individuals and machines as part of their network infrastructure. The availability of data generated by people and machines brings about an opportunity to compile context awareness. In this light, a new information technology paradigm must be proposed to consider the rationality and irrationality of human behavior when managing the resources of underlying infrastructures. For instance, to suppress the data uncertainty that humans add to emerging networks, the structuralist nature of psychoanalysis can be researched to model human uncertainty.

## 7. Conclusions

It is reasonable to assume that human-centric networks have a great impact on people’s lives. However, the intensive use of HCNs give rise to a huge amount of generated data traffic consuming a large quantity of DCN resources in terms of processing power, storage capacity, and energy. However, these data represent the raw material from which important information is derived to be used by new disruptive applications.

In this work, we presented a quite comprehensive review of decision-making computational methods such as MCDM, optimization algorithms, and machine learning that have been proposed to be used in different applications domains, for example, telecommunications, healthcare, transport and logistics, business and investment decisions, and industrial production planning. It has been shown that all of the proposed methods can be severely impaired by the uncertainty present in extracted data that may corrupt the information derived from them. Uncertainty is very difficult to deal with because of the diversity of sources ranging from electrical noise to human behavior. Clearly, a new multidisciplinary computational paradigm must be developed to assess and address uncertainty factors.

Data security and privacy are of paramount importance to be taken into account due to the implications and consequences of improper use of sensitive personal, technical, and business information. A strong emphasis must be put on promoting the establishment of governance rules to prevent abuse of sensitive data to protect society in all its dimensions. Otherwise, HCN will loose credibility and will not be accepted and adopted by anyone.

A closer look at the emerging concepts as Smart Spaces or Industry 4.0 reveals a worrying lack of worldwide accepted standards, even at the level of the concept definitions, that may jeopardize interoperability of devices, and single- and cross-domain applications.

## Figures and Tables

**Table 1:** Grouping of semantic data-mining work.

Work Area	Related Work	Key Points
IoT	[12,13,14,15,16,17,18,19]	Interoperability Data sources
Semantic Web	[11,20,21,22,23,24,25]	Association rules mining Ontological data Recommender systems Knowledge extraction
Industry 4.0	[26,27]	Data marketplace Logistics infrastructure

**Table 2:** MCDM method review.

Method	Related Work	Key Points
ELECTRE	[35,36,37]	Execution time optimization
PROMETHEE	[38,39,40,41]	Infrastructure construction Energy sector Engineering decision problems
AHP	[42,43,44]	Decision-making under uncertainty Recommendation systems
VIKOR	[45,46,47]	Sentiment analysis in social networks Performance evaluations
TOPSIS	[48,49,50]	Several combinations of methods
DEA	[51]	Evaluate relative efficiency

**Table 3:** Review of optimization methods.

Method	Related Work	Key Points
Fuzzy logic	[38,45,58,59,60,61,62,63,64,65,66,67,68,69,70,71]	They handle diverse types of uncertainties
Game theory	[72,73,74,75]	Decision learning Decision-making
Bayesian networks	[76,77,78,79,80]	Decision-making under uncertainty
Stochastic process	[6,81,82]	Adaptive systems Context-aware decision process
Support vector machine	[83,84]	Sustainability indicators

**Table 4:** Works that apply supervised learning.

Work Area	Related Work	Key Points
Telecommunications	[7,87,88,89,90,91,92]	Self-Organizing Network Improve QoS Virtual machine migration over WAN links 5G auto-configuration
Energy	[93,94,95,96]	Operations planning Behaviors nuclear energy system Power grid
Transport	[97,98,99,100,101]	Lane change Driving on rough terrains Robot mobility
Enhance decisions	[102,103,104,105]	Complex negotiations Combination of techniques Support for human decisions
Complex systems	[106,107,108,109]	Human behavior
Optimization problems	[84,110,111,112,113,114]	Deal with uncertainty

**Table 5:** Works supported by unsupervised learning.

Work Area	Related Work	Key Points
Transport	[115,116,117]	Overcoming obstacles
Health	[118,119,120]	Improve diagnostic accuracy Decrease diagnostic times
Business decisions	[83,121,122,123,124]	Improve profits
Deal with uncertainty	[125,126,127,128,129]	Battlefield decision-making Geology decisions Gambling

**Table 6:** Studies supported by reinforcement learning.

Work Area	Realted Work	Key Points
Telecommunications	[130,131,132]	Resource management 5G Routing
Energy	[133,134]	Supply and demand balance
Transport	[135,136,137,138,139,140]	Autonomous driving Driving experience
Optimization problems	[141,142,143,144,145]	Noisy data Scalable solutions Data fitting Computational costs

**Table 7:** Studies supported by deep learning.

Work Area	Realted Work	Key Points
Telecommunications	[146,147,148,149,150]	Resource allocation SDN
Human behavior	[151,152,153]	Behavior modeling Decision-making from psychology
Uncertainty	[154,155,156,157,158,159,160,161,162]	Noisy or incomplete data Decision-making under uncertainty

**Table 8:** Car accident scenario.

User 1	User 2
I’m in the middle of an accident Everyone is dead Photo: Selfie I should have been in the shopping mall, not here	There has been an accident There are many injured people Photo: showing several cars in the accident Please send some help

**Table 9:** Example of Lacan’s and Bion’s indices assignment.

	User 1						User 2					
Piece of user data	$I_{m}$	$I_{u}$	$I_{h}$	$I_{a}$	α	β	$I_{m}$	$I_{u}$	$I_{h}$	$I_{a}$	α	β
1	0.5	0.0	0.5	0.0	1	0	0.0	0.5	0.5	0.0	1	0
2	0.1	0.0	0.9	0.0	0	1	0.0	0.3	0.7	0.0	1	0
3	0.0	0.0	1.0	0.0	0	1	0.0	0.5	0.5	0.0	1	0
4	0.0	0.0	1.0	0.0	0	1	0.0	0.2	0.8	0.0	1	0
mean E	0.15	0.0	0.85	0.0	0.25	0.75	0.0	0.375	0.625	0.0	1	0

## Data Availability

No new data were created or analyzed in this study. Data sharing is not applicable to this article.
